# Decreased Usage of Specific Scrib Exons Defines a More Malignant Phenotype of Breast Cancer With Worsened Survival

**DOI:** 10.1016/j.ebiom.2016.05.009

**Published:** 2016-05-07

**Authors:** Gergana Metodieva, Samson Adoki, Berthold Lausen, Metodi V. Metodiev

**Affiliations:** a School of Biological Sciences, University of Essex, CO4 3SQ, United Kingdom; b Department of Mathematical Sciences, University of Essex, CO4 3SQ, United Kingdom

**Keywords:** SCRIB, Breast cancer, Splicing, Survival, Mitosis, Polarity

## Abstract

SCRIB is a polarity regulator known to be abnormally expressed in cancer at the protein level. Here we report that, in breast cancer, an additional and hidden dimension of deregulations exists: an unexpected SCRIB exon usage pattern appears to mark a more malignant tumor phenotype and significantly correlates with survival. Conserved exons encoding the leucine-rich repeats tend to be overexpressed while others are underused. Mechanistic studies revealed that the underused exons encode part of the protein necessary for interaction with Vimentin and Numa1, a protein which is required for proper positioning of the mitotic spindle. Thus, the inclusion/exclusion of specific SCRIB exons is a mechanistic hallmark of breast cancer, which could potentially be exploited to develop more efficient diagnostics and therapies.

## Highlights

• Proteome analysis of breast tumors shows loss of C-terminal Scribble sequences.
• Distinct pattern of SCRIB exon usage revealed by tumor RNA-seq analysis.
• SCRIB exon usage patterns correlate with long-term survival in breast cancer.
• Human Scribble interacts with Numa1, a protein which regulates the positioning of the mitotic splindle.

We report experimental evidence showing that human breast tumors exhibit a distinct SCRIB exon usage preference that leads to overexpression of conserved N-terminal domains and loss of C-terminal sequences that are involved in the fine tuning of the cell cycle. This is specific for breast tumors and could potentially open new ways for developing stratified therapies for breast cancer.

## 1 Introduction

In epithelial cells planar polarity is maintained by proteins that are encoded by tumor suppressor genes such as SCRIB (Bilder et al., 2000, Bilder and Perrimon, 2000). Scribble, the protein product of SCRIB, is crucial for the proper maintenance of epithelial cell integrity and function (Zhan et al., 2008); it is required for E-cadherin-mediated cell-cell adhesion and, when its expression is down-regulated, epithelial cells acquire mesenchymal appearance and their migration is augmented (Qin et al., 2005). In epithelial cells the orientation of the mitotic spindle is restricted to the plane of the epithelium to ensure that daughter cells will remain within the layer. It was shown recently that Scribble is involved in this process and when its expression is knocked down in Drosophila the orientation of the mitotic spindle becomes random. When combined with inhibition of apoptosis, Scribble knock-down is sufficient to induce epithelial to mesenchymal transition and formation of tumor-like structures (Nakajima et al., 2013). Scribble expression and localization is frequently deregulated in human cancers, including breast cancer, where it is either lost or abnormally overexpressed and localized (Zhan et al., 2008, Feigin et al., 2014). In this study we show that, in human breast cancer, the expression of several exons, which share very little similarity with the sequence from Drosophila, is decreased relative to the exons encoding the rest of the protein. Furthermore, this pattern of exon usage correlates with long-term survival. The underused exons encode part of the C-terminal proline-rich domain of Scribble, which we found to become increasingly phosphorylated in mitosis, leading to association with Numa1, a protein known to be crucial for the proper positioning of the mitotic spindle in polarized cells.

## 2 Materials and Methods

### 2.1 Reagents

Chemicals and HPLC solvents, unless indicated otherwise in the text, were purchased from Thermo Fisher. The highest available grades were used. The following antibodies were used for Immunoprecipitation and indirect immunofluorescence: mouse monoclonal anti-GFP (B-2, Santa Cruz, CA), rabbit polyclonal anti-Numa1 (H300, Santa Cruz, CA), mouse monoclonal anti-Scribble (C6, Santa Cruz, CA), goat anti-mouse Alexa Fluor 467 (Invitrogen, USA), goat anti-rabbit Texas red-conjugated (Jackson IR laboratories). Protein A/G coated magnetic microparticles (Thermo Fisher). MS-grade trypsin was used for protein digestion (Promega).

### 2.2 Plasmids and Mutagenesis

The GFP-Scribble sequence cloned in pEGFP-N1 (Clonthech) used to generate Scribble mutants was a generous gift from Professor Ian Macara, Vanderbilt University School of Medicine. The following oligonucleotides were used to change S1448 to A and D, and to delete the C-terminus: GCTGGAGGGAAGATGGCTTAATCTCCCTGCTCCCCTAG to change codon 1293 to TAA for stop; AGGCAGAGCCCGGCGGCCCCCCCGCCCCTGGGA to change codon 1448 to GCC for alanine; AGGCAGAGCCCGGCGGACCCCCCGCCCCTGGGA to change codon 1448 to GAC for aspartate. Mutagenesis was confirmed by sequencing and mass spectrometry at the protein level.

### 2.3 Cell Culturing and Transfection

HEK293T cells were grown on DMEM medium supplemented with 10% FCS at 37 °C and 5% CO_2_ and split every third day. For confocal imaging the cells were transfected in in Nunc™ Lab-Tek™ chamber slides or on coverslips in 24 well dishes. For co-IP experiments the cells were transfected in 10 cm dishes. In both cases the transfection was with TurboFect (Thermo Fisher) according to the manufacturer's instructions.

### 2.4 Immunoprecipitation

Cells were harvested, washed with PBS and lysed with buffer containing 1% Igepal 40, 50 mM Tris-HCl, pH 6.8, protease and phosphatase inhibitors. Lysates were cleared by centrifugation and incubated with 4 μg anti-GFP antibody (B-2, Santa Cruz Biotechnology) or anti-Scribble antibody (C-6, Santa Cruz Biotechnology) for 45 min at RT on a rotator. 20 μl magnetic protein A/G microbeads (Thermo Fisher) were added and incubated for 1 h at RT on the rotator. Beads were washed 3 × with 1 ml PBS containing 0.1% Tween 20 and once with 1 ml PBS without detergent. The Magnarack system was used to pellet the magnetic microbeads between washes. After the removal of the final wash volume 20 μl of trypsin solution (30 mg/ml trypsin in 25 mM NH_4_HCO_3_ containing 1 M urea) was added to the microbeads and the digestion was carried out at 30 °C for 16 h.

### 2.5 Nano-scale LC-MS/MS and Analysis of MS/MS Data

The analysis of protein digests by electrospray ionization MS was performed using a hybrid LTQ/Orbitrap Velos instrument as previously described (Greenwood et al., 2012, Metodieva et al., 2013, Croner et al., 2014). MaxQuant was used to analyze MS/MS data to identify and quantify proteins (Cox and Mann, 2008).

### 2.6 Identification of Co-precipitated Proteins

To evaluate the statistical significance of the IP-LC/MS results we used the spectral counts for GFP, Scribble and co-precipitated proteins and computed p-values using two different tests: the G test and Chi squared test. For the Chi squared test we constructed contingency tables of observed and expected counts. The expected values for each candidate protein were derived from either the observed Scribble counts or the observed GFP counts. This analysis was performed in R.

### 2.7 Targeted Detection of Numa1

A SRM scan was added to the LC-MS/MS method to isolate and fragment the doubly-charged Numa1 peptide AALMESQGQQQEER at *m*/*z* of 802.87. The precursor was accumulated for 100 ms in the linear ion trap and then fragmented by collision-induced dissociation. The intensity of the y7 fragment ion (*m*/*z* = 874.45) was used for quantification. Label-free quantitation was performed by integrating the ion intensities as described previously (Metodieva et al., 2009).

### 2.8 Calculation of Relative Phosphorylation Site Occupancy Rates

We followed the approach described in Olsen et al. for SILAC-labelled peptides (Olsen et al., 2010). The occupancy rate calculation as described in Olsen et al. requires SILAC labeling. Here we modify the procedure to allow relative occupancy rate calculation from label-free experiments provided there exist co-eluting peptides derived from the same protein that can serve as internal normalizing standards. The procedure as used to analyze S1448 phosphorylation dynamics during the cell cycle is explained below.

Two co-eluting unmodified Scribble peptides were used to normalize the ion current for the phosphopeptide and its unmodified counterpart: RLPDGIGQLK for the phosphopeptide QSPAS (ph)PPPLGGGAPVR and GHAGNPRDPTDEGIFISK for QSPASPPPLGGGAPVR. E_0_ is ionization efficiency of base peptide; E_1_ is ionization efficiency of mod peptide; I_0_ is ion intensity of base peptide; I_1_ is ion intensity of mod peptide; E_2_ is ionization efficiency of normalizing peptide co-eluting with base peptide; E_3_ is ionization efficiency of normalizing peptide co-eluting with mod peptide; I_2_ is ion intensity of normalizing peptide co-eluting with base peptide; I_3_ is ion intensity of normalizing peptide co-eluting with mod peptide. To calculate molar ratio (mod/base) we assume concentration (M) = I ∗ E so mod/base = I_1_E_1_/I_0_E_0_.

Since ionization intensities are unknown we use control/treatment ratios to write equation and eliminate E:

We assume that the concentrations of the normalizing peptides do not change relative to the protein concentration because they do not undergo modification under cell cycle control. The ratio I_1_E_1_/I_3_E_3_ is run-invariant for a given biological replicate because the two peptides co-elute so any fluctuations in spray conditions would affect them equally and we assume that ionization efficiencies depend only on the sequence. On the other hand this ratio would be changed in response to treatment if the phosphorylation site occupancy is treatment-dependent. It follows then that:

(1) $$\frac{I_{1}^{t}}{I_{3}^{t}}/\frac{I_{1}^{c}}{I_{3}^{c}}=\frac{\left\lfloor {\mathrm{mod}}^{t}\right\rfloor }{\left\lfloor {\mathrm{mod}}^{c}\right\rfloor }=X$$

(2) $$\frac{I_{0}^{t}}{I_{2}^{t}}/\frac{I_{0}^{c}}{I_{2}^{c}}=\frac{\left\lfloor {\mathrm{base}}^{t}\right\rfloor }{\left\lfloor {\mathrm{base}}^{c}\right\rfloor }=Y$$

From equations 1 and 2 we now calculate the site occupancy rates in control and treatment to be related as follows:

(3) $$\frac{\left[{\mathrm{mod}}^{t}\right]}{\left\lfloor {\mathrm{base}}^{t}\right\rfloor }=\frac{X}{Y}\left(\frac{\left[{\mathrm{mod}}^{c}\right]}{\left[{\mathrm{base}}^{c}\right]}\right)$$

(4) $$\frac{a}{b}=\frac{X}{Y}$$

Where *a* is the occupancy rate $\frac{\left[{\mathrm{mod}}^{t}\right]}{\left\lfloor {\mathrm{base}}^{t}\right\rfloor }$ and *b* is the occupancy rate $\frac{\left[{\mathrm{mod}}^{c}\right]}{\left[{\mathrm{base}}^{c.}\right]}$

The ratios from Eq. 4 are then used to graph the corresponding data points for the different phases of the cell cycle assuming G1-S as a baseline.

### 2.9 Confocal Microscopy

Cells were seeded on Nunc™ Lab-Tek™ chamber slides or on coverslips in 24 well plates at 30%–50% confluency. The next day they were washed with PBS and fixed with 4% formaldehyde for 15 min RT. They were washed with PBS and permeabilized with 0.5% Triton X-100 in PBS for 5 min and blocked for 1 h with 5% BSA in PBS. Primary antibody was added in 5% BSA, at 1:100 dilution for 1 h. Samples were washed 3 times with 1% BSA in PBS with 0.1% Tween 20. Secondary fluorophore – conjugated antibody was applied for 1 h and after 3 × washing with PBS – 0.1% Tween 20, samples were stained with DAPI for 15 min and washed with PBS. Coverslips were mounted on glass slides and chamber slides had coverslips mounted using Vectashield (Vector Laboratories, Burlingame). For image acquisition, a Nikon A1Si confocal microscope was used with a plan-apochromatic VC 1.4 NA 60 × magnifying oil-immersion objective. Images were acquired in three or four channels, using one-way sequential line scans. DAPI was excited at 405 nm with laser power 3.0, and its emission collected at 450/50 nm with a PMT gain of 104. Alexa Fluor 496 was excited at 488 nm with laser power 5.6, its emission collected at 525/50 nm with a PMT gain of 85. Texas red was excited at 561 nm with laser power of 5.8, and collected at 595/50 nm with a PMT gain of 139. Cy 3 was excited at 560.5 with laser power of 1.6 with a PMT gain of 84. The pinhole size was 42.5 μm. Scanner zoom was centered on the optical axis and set to a lateral magnification of 55–90 nm/pixel. Axial step size was 500 nm, with 15–30 image planes per *z*-stack. Image analysis was performed on NIS-Elements (version 3.21.03, build 705 LO).

### 2.10 Wound-healing Assay

The cells were transfected in 24 well plate with 4 replicates per transfection. After 16 h each well was wounded using yellow tip pipette and carefully washed with complete medium. The plate was imaged at 0 h, 24 h and 48 h time points using Nikon Ti-E Wildfield inverted microscope and data processing and statistical analysis was done using Nikon Elements software.

### 2.11 Flow Cytometry

The cells were harvested as a single cell suspension and fixed to anchor the GFP with 0.5% paraformaldehyde for 15 min at RT. The cells were then washed twice in wash buffer (PBS + 0.1% bovine albumin). Aliquots of 1 ml (1–2 × 106 cells/ml) each were placed in 15 ml polypropylene V-bottomed tubes on ice and allowed to cool. A 3 ml cold (− 20 °C) absolute ethanol was added dropwise while vortexing to minimize clumping and the tubes were incubated at least 1 h at − 20 °C. After that the cells were centrifuged and washed with PBS. Propidium iodide (PI) staining solution was prepared with 3.8 mM sodium citrate and 40 μg/ml PI in PBS and 1 ml was added to the cell pellet and mixed well. Next, 50 μl of 10 μg/ml RNase A stock solution was added and the cells were incubated for 1 h at RT.

Stained samples were analyzed on a BD Accuri instrument and the data was processed using FCS Express 4 software.

### 2.12 RNA-seq Data Analysis

To quantify exon expression RNA-seq data were downloaded from TCGA (http://cancergenome.nih.gov/), patient information is presented in Table 1, and analyzed in R: First, the 1095 breast tumor RNA-seq data files containing “exon quantification” in their names were imported and Scribble exon entries were extracted and merged into a single data frame. Next, this data frame was merged with a table containing the clinical data using patient barcode as identifier for merging. Since significant number of records had zero or very short follow-up time, the data was censored to contain patients with at least 90 days follow-up. We followed the normalization approach implemented in DEXSeq (Anders et al., 2012) to assess exon usage: SCRIB exons expression data was normalized by dividing each exon RPKM count by the sum of counts of all other exons for each patient. Descriptive statistics (boxplots) were then used to examine the difference in exon usage between different groups of cancer samples. For survival analysis breast cancer patients were clustered, initially in 4 clusters, using the k-means algorithm. Clusters showing nearly identical survival curves were merged. Kaplan-Meier survival curves were calculated using the classification obtained by clustering. Corresponding p-values were computed using the log rank test.

The analysis of RNA-seq datasets from MCF10A breast cancer cells (SRA study ERP007109 accessible at http://www.ncbi.nlm.nih.gov/sra/?term=ERX593981) was performed as follows:

Aligned data for 16 RNA-seq runs were from ftp://ftp.sra.ebi.ac.uk/vol1/fastq/ERR637; the reads aligned to the SCRIB gene region were extracted directly from the repository using the sam-dump utility of the sra-toolkit suite. The RPKMs for each exon and for each file were then calculated using the total number of aligned reads as shown on the SRA repository webpage, the number of reads mapping to each exon and the length of the exons in kilobase. The following commands were used to retrieve the data and write them to a file:

sam-dump –aligned-region chr8:143790920-143815379 $SRA_run_name > $name.sam

samtools view -bS $name.sam > $name.bam

samtools sort $name.bam > -o $name.sorted.bam

samtools index $name.sorted.bam

bedtools multicov -bams $file1 $file2 ... -bed $name.bed > $name

The bed file with the exon coordinates was generated using the table browser tool from UCSC Genome Browser available at http://genome.ucsc.edu/cgi-bin/hgTables.

The R code shown in the iPython notebook “scrib_revised.html” can be used to repeat the survival analysis and plot the exon usage boxplots using the datasets “data_full_aggregated.csv” and “MCF10A.csv” available in the Supplementary Information online.

## 3 Results

### 3.1 Scribble Expression in Breast Tumors Analyzed by High-resolution LC-MS/MS: Breast But not Colorectal Tumors Express Predominantly Truncated Scribble Protein Lacking the C-terminal Proline-rich Domain

In an ongoing study, in which we profile the membrane proteome of frozen tumors by high-resolution mass spectrometry(Alldridge et al., 2008, Greenwood et al., 2012, Metodieva et al., 2013, Croner et al., 2014), we identified an unexpected pattern of Scribble expression: in breast tumors Scribble is detected with multiple peptide MS/MS spectra but most of the detected peptides are derived from the highly conserved exons encoding the N-terminal leucine-reach repeat domain. This is not the case when cultured non-cancer cells are analyzed and in colorectal tumors where C-terminal peptides are detected frequently. These results are presented in Supplemental Figure 1 and Supplemental Table 1. It appears that breast tumors tend to express truncated Scribble isoforms, despite the fact that constitutive truncation of the C-terminal part of Scribble was found to be embryonic lethal in the mouse (Murdoch et al., 2003).

### 3.2 Statistical Analysis of SCRIB Exon Usage Using RNA-seq Data From The Cancer Genome Atlas Project (TCGA): Breast But Not Colon Tumors Overuse Exons Encoding the N-terminal Part of the Protein and Underuse Exons Encoding the C-terminal Part

**Fig. 1:**
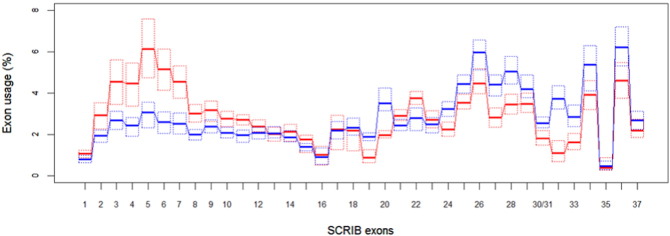
Breast tumors overuse SCRIB exons 1:11 and underuse exons 30:37 compared to colon tumors. Exon usage was assessed using RNA-seq data from TCGA. The relative exon usage is given with boxplots omitting the whiskers. Quartiles are denoted with dotted lines. Breast cancer data (n = 1095) is shown in red and colon cancer data (n = 458) is shown in blue. The exon usage for each exon was calculated by dividing the RPKM count by the sum of counts for all other exons and multiplying by 100.

To further investigate the underlying causes for this unusual pattern of Scribble protein expression in breast tumors and test its clinical significance, we carried out statistical analysis of the available large-scale breast cancer sequencing data and the associated clinical information. Quite intriguingly, we found that breast tumors have a distinct SCRIB exon usage profile when compared to colon tumors. This is shown on Fig. 1. In particular exons 2 to 11 are over used by breast tumors and exons 24 to 34, 36 and 37 are underused when compared to colon tumors. This pattern of exon expression suggests that the increased detection of N-terminal Scribble peptides is due to overexpression of transcripts containing the N-terminal exons but missing some of the exons encoding the C-terminal half of the protein.

**Fig. 2:**
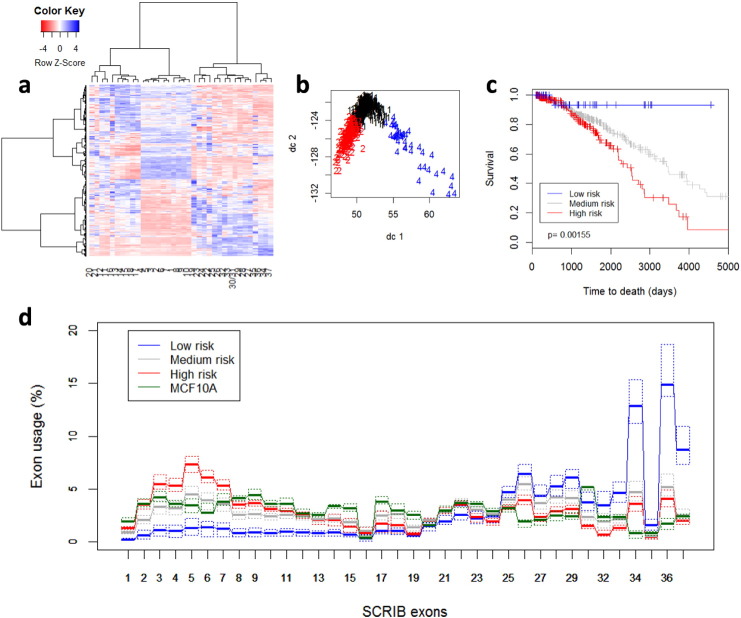
Scribble exon usage patterns correlate with survival of breast cancer patients (n = 877). a: Heatmap of exon usage generated with the R package heatmap.2 using Ward's algorithm for hierarchical clustering; b: Cluster plot using the R package Cluster and its function clusterplot. Tumors were classified into 3 clusters based on exon usage and survival rate as detailed in Material and Methods. The plot uses the first 2 principal components indicated as dc1 and dc2; c: Kaplan-Meier survival curves based on clustering by SCRIB exon usage data. Unsupervised K-means clustering was applied to group the patients into 3 clusters. The indicated p-value was calculated using the log-rank test on 2 degrees of freedom; d: SCRIB exon usage (in percent) by cluster and by MCF10A cells. The colors corresponds to the Kaplan-Meier curves in a. Boxplots are plotted as in Fig. 1. Thick lines indicate the median and quartiles are plotted with dotted lines.

In addition, survival analysis showed that overuse of exons 4, 5, and 6 and underuse of exons 32, 33, and 34 significantly correlates with survival. This is shown in Fig. 2: we used unsupervised K-means clustering to group all cases into 3 clusters based on normalized SCRIB exons usage, which was computed by dividing the counts for each exon to the sum of counts of all other exons for each sample. Cluster membership was then used to construct the Kaplan-Meier survival curves shown in Fig. 2c and the boxplots in Fig. 2d. It is quite evident that the group characterized by increased expression of exons 2:11 (red) has a significantly worse survival than the group with high expression of exons 30:37 and low expression of exons 2:11 (blue). Fig. 2d also shows the SCRIB exon usage of normal breast MCF10A cells (green), which we computed from 16 RNA-seq experiments examining gene expression during acinus formation in 3D cultures in matrigel. MCF10A cells exhibit SCRIB exon usage patterns which are similar to the group of breast tumors characterized by medium survival outcomes and depicted by dark-grey boxplots in Fig. 2d.

**Fig. 3:**
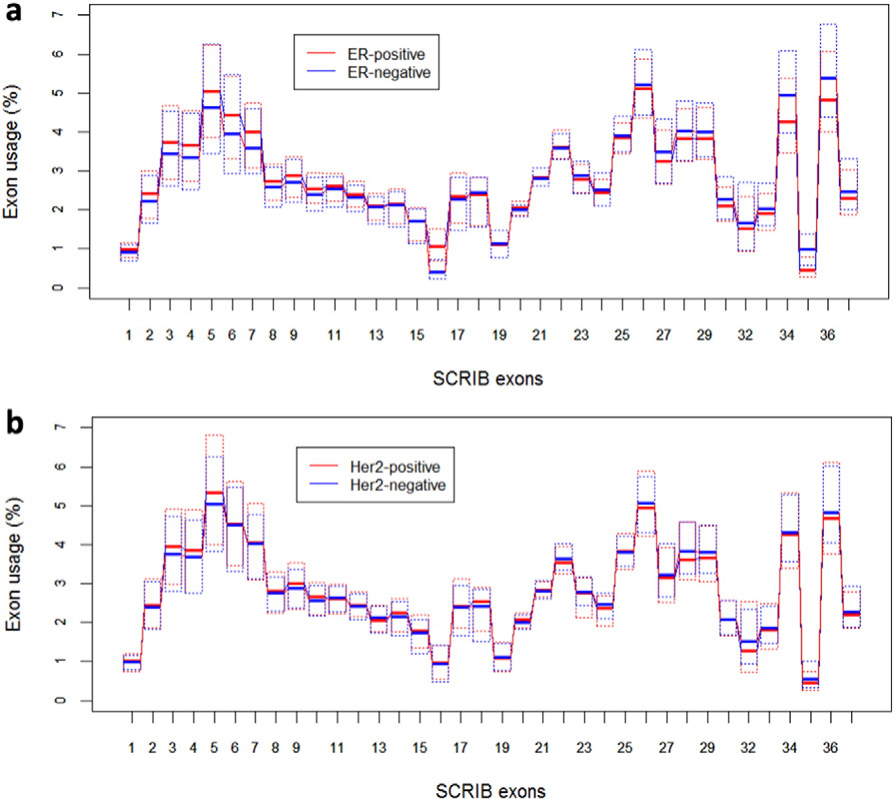
Statistical analysis of Scribble exon usage in ER-positive (n = 674) and ER-negative (n = 189) (top) and Her2-positive (n = 116) and Her2-negative (n = 465) breast tumors (bottom). The exon usage was calculated from RNA-Seq data as described in Materials and Methods.

Fig. 3 shows results from statistical analyses of the effect of estrogen receptor (ER) and Her2 status as obtained by histochemical assays, on the pattern of SCRIB exon usage in the studied breast tumors. There is no apparent effect of the Her2 status and there is a significant effect of the ER status on the usage of 4 exons: exons 34, 35 and 36 are included in SCRIB transcripts with higher frequencies in ER-negative tumors than in the ER-positive tumors. Exon 16 is included in SCRIB transcripts with a higher frequency in ER-positive tumors. The corresponding p-values calculated by the non-parametric Wilcoxon test and adjusted for multiple testing using the Holm procedure are 7.52e-33 for exon 16, 6.50e-05 for exon 34, 1.49e-20 for exon 35, and 4.65e-04 for exon 36.

Taken together the results reported in this section show that SCRIB exon usage correlates with overall survival in breast cancer. SCRIB exon usage patterns calculated from RNA-seq data show significant correlation with ER status but not Her2 status as determined by immunohistochemical methods.

### 3.3 Mechanistic Studies in HEK293 Cells: The C-terminus of Scribble is Increasingly Phosphorylated in Mitosis and is Required for Interactions with Numa1 and Vimentin

To find out how the apparent underuse of C-terminal exons affects the function of Scribble we performed protein interaction experiments using wild-type Scribble and various mutants including a truncated form of the protein lacking exons 30–37 as baits. The obtained results showed that the C-terminus is required for association with Vimentin and, surprisingly, with proteins involved in the regulation of the mitotic spindle orientation, notably the nuclear mitotic apparatus protein Numa1 (see Supplemental Table 2). The C-terminus is not required for the association with Beta-pix and Git1, which were previously shown to form a tight complex with Scribble (Audebert et al., 2004).

**Fig. 4:**
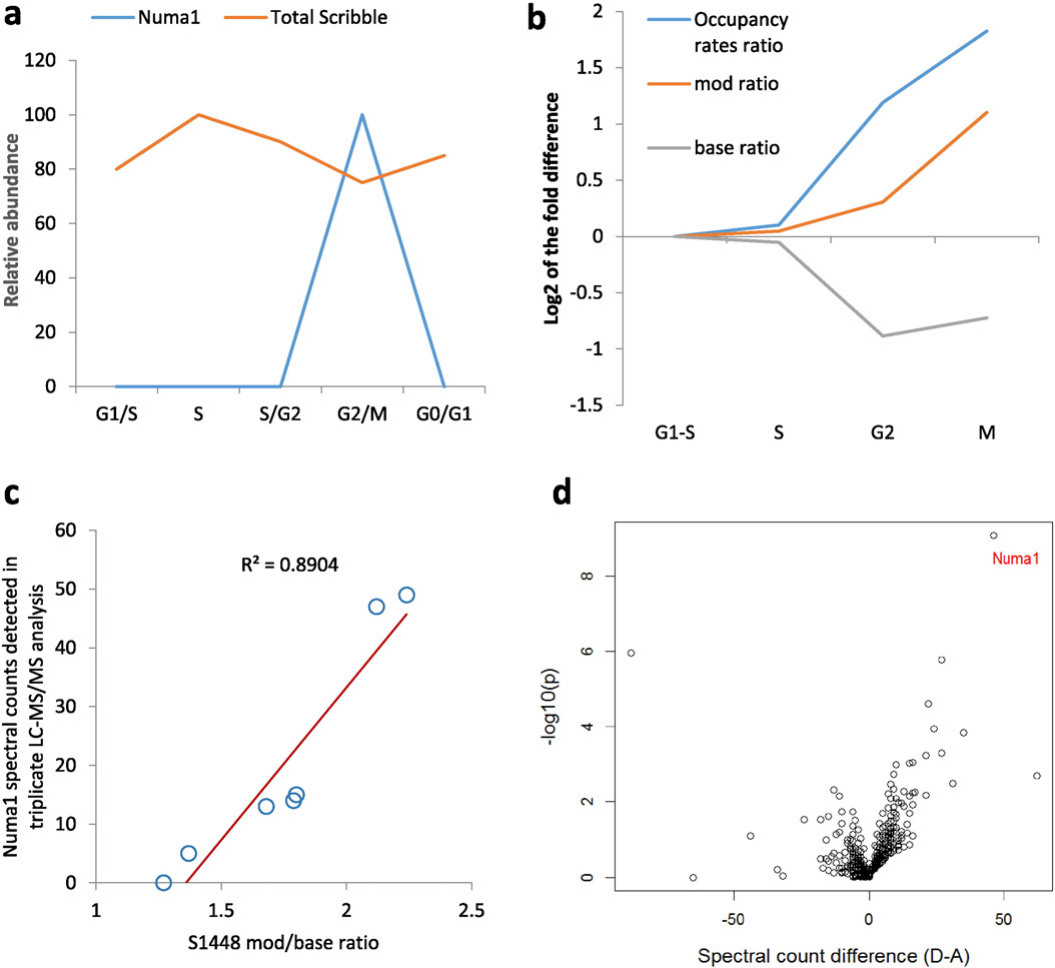
The amount of Numa1 in GFP-Scribble immunoprecipitates correlates with the phosphorylation state of S1448. a: Detection of Numa1 in Scribble immunoprecipitates from cells in different phases of the cell cycle. Numa1 was quantified by targeted mass spectrometry as described in Materials and Methods; b: Scribble S1448 phosphorylation occupancy rates through the cell cycle of HEK293 cells; c: The amount of Numa1 in GFP-Scribble pull-down depends on S1448 phosphorylation. Data is from 7 independent GFP-Scribble IP-LC/MS experiments, each analyzed in triplicate LC-MS/MS runs. The total number of Numa1 spectral counts detected in each experiment is plotted against the ratio of intensity of the Scribble peptide phosphorylated on S1448 to the intensity of the unphosphorylated peptide; d: Volcano plot of data obtained in IP-LC/MS experiments using S1448A and S1448D mutants of Scribble as baits.

Furthermore, the analysis of the obtained results showed that Numa1 is consistently detected in the Scribble pull-down only if a specific Scribble phosphorylation site, S1448, is highly phosphorylated. This is shown in Fig. 4c. Notably, S1448 resides in a part of the protein which is encoded by exon 32, one of the exons strongly associated with survival and underused in breast tumors. Scribble S1448 phosphorylation has been reported in several previous studies (Beausoleil et al., 2004, Olsen et al., 2006, Nagasaka et al., 2010) and we have recently reported that S1448 is differentially phosphorylated in response to CD74 overexpression, a phenomenon frequently observed in cancer (Metodieva et al., 2013). To further understand the role of this phosphorylation, we generated phospho-mimetic and phosphorylation-deficient mutants by substituting S1448 with aspartic acid and alanine. These were then used in protein-interaction screens along with wild type Scribble. The results from these screens are summarized in Table 2 and in Fig. 4d. Numa1 was the top protein to show statistically-significant difference in preference to scrib^S1448D^ over scrib^S1448A^ as bait. To obtain further evidences that S1448-phosphorylated Scribble associates with Numa1 we carried out IP-LC/MS experiments using cell cycle- synchronized cells and endogenous Scribble as bait. As shown in Fig. 4a, wild type Scribble associated with Numa1 only in cells arrested by nocodazole, which was coincident with the peak of S1448 phosphorylation. In these experiments S1448 behaved as a typical mitotic phosphorylation site. Its occupancy increases 3.5-fold from G1/S to M (Fig. 4b).

**Fig. 5:**
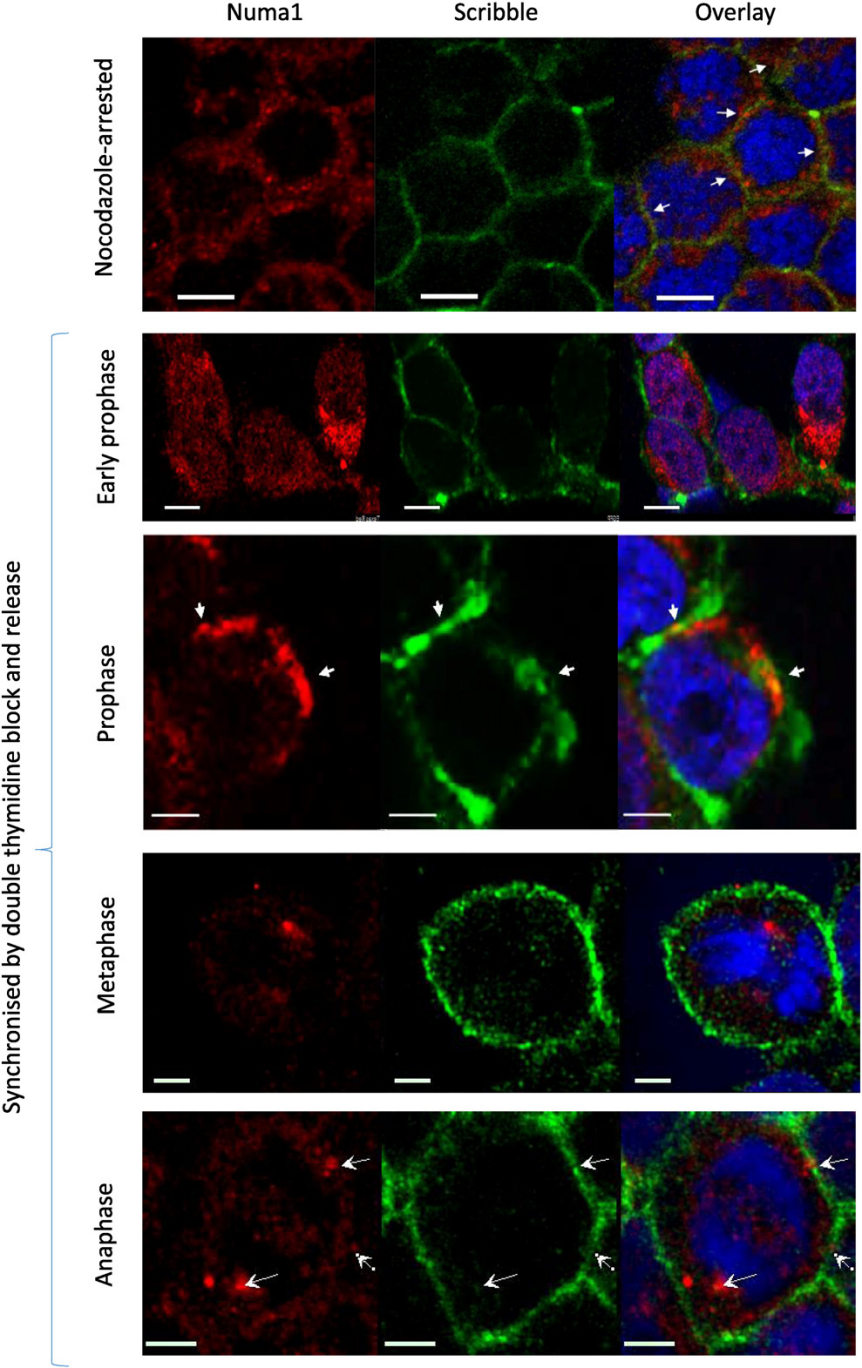
In mitotic cells Numa1 exits the nucleus and colocalizes with Scribble in prophase. Scribble (green) and Numa1 (red) were visualized by indirect immunofluorescence and confocal microscopy in mitotic HEK293. DNA (blue) was visualized with DAPI. Top: nocodazole-arrested cells stained with Cy3-labelled secondary antibody (Numa1) and Alexa Fluor 496 –labelled secondary antibody (Scribble). Bottom: cells were synchronized by double thymidine block, released and processed at appropriate time points. Here Numa1was labelled with Texas Red and the images were acquired separately for Texas Red and Alexa Fluor 496 to rule out possible bleed through phenomena. The arrows in the images of cells in prophase point to area of cortical colocalization of the two proteins. The arrows in the bottom row of images point to the spindle pole and cortical patches where Numa1 and Scribble colocalize. The size bar is 10 μm for the images on the top and 5 μm for the images on the bottom.

Fig. 5 presents results from another set of experiments utilizing indirect immunofluorescence. It shows that Scribble and Numa1 colocalize in mitotic cells: in HEK293 cells synchronized by double thymidine block or nocodazole arrest the two proteins colocalized at the cell cortex further corroborating the results obtained by immunoprecipitation and mass spectrometry.

Finally, expression of the phosphorylation-deficient S1448A mutant augmented migration and would healing in HEK293 cells but caused more-pronounced G1 arrest as compared to a control expressing wild type Scribble. In contrast, expression of the phospho-mimetic mutant strongly inhibited migration and wound healing and increased the proportion of cells arrested in G2/M (Supplemental Fig. 2 and Supplemental Table 3).

Thus, the results obtained in IP-LC/MS experiments, confocal microscopy and wound healing, and flow cytometry assays show that Scribble becomes increasingly phosphorylated on S1448 in mitosis correlating with interaction with Numa1 at the cell cortex. On the other hand expression of a phosphorylation deficient S1448A mutant induced G1 arrest in HEK293 cells but increased their migration and augmented wound healing.

## 4 Discussion

The homolog of the human tumor suppressor SCRIB was first characterized in Drosophila (Bilder et al., 2000). The product of human SCRIB, Scribble, is a multidomain protein acting as scaffold involved in multiple signaling pathways. Scribble is deregulated at various levels in human cancer. It was found to be lost or mislocalized in some breast tumors (Zhan et al., 2008). It can be also overexpressed and mislocalized in breast cancers with decreased expression of miR-296 (Savi et al., 2014) and overexpressed and mislocalized in many cancer cell lines and diverse tumors (Vaira et al., 2011).

Human Scribble shares significant degree of sequence homology with Drosophila LAP4, but this is only found in the N-terminal part of the protein containing the leucine-rich repeat domain, and the downstream PDZ domains. The C-terminal proline-rich part of human Scribble shares very little sequence similarity with Drosophila LAP4. Here we show by statistical analysis of RNA-seq data that the exons encoding this C-terminal proline-rich domain are underexpressed in high-risk breast cancers. Membrane proteome analysis of an independent tumor cohort further corroborated the statistical analysis showing that in breast tumor tissue Scribble is detected by LC-MS/MS predominantly by peptides derived from the N-terminal part of the protein. Finally, pull-down experiments and phosphoproteomic screens led to the discovery that human Scribble interacts with specific mitotic proteins in a phosphorylation-dependent manner. As shown in Fig. 4, the amount of Numa1 in the GFP-Scribble pull-down was highly correlated with the phosphorylation occupancy rate of S1448. Additionally, S1448A mutants of Scribble failed to precipitate Numa1 while when S1448D mutants were used as baits Numa1 was co-immunoprecipitated. This result can be interpreted in two possible ways: either phosphorylated S1448 is a structural determinant of the interaction interface, or phosphorylation of this residue is required for progression of the cell cycle to a phase where Numa1 can exit the nucleus and associate with Scribble. In both cases we can assume that the interaction of the two proteins depends on S1448 phosphorylation.

Interestingly, the overexpression of the S1448A mutant augmented migration and wound healing but caused more pronounced G1 arrest compared to overexpression of wild type Scribble. In contrast, the S1448D mutant strongly inhibited wound healing while allowing more cells to progress to G2//M. These results appear to support recent reports suggesting that Scribble might have a dualistic role in cancer depending on mechanistic context: it can act as tumor suppressor but it can also promote tumor progression and metastasis through its interactions with PAK, NOS1AP and SHOC2 (Anastas et al., 2012, Nola et al., 2008, Young et al., 2013).

Taken together, our results outline a possible model, in which the C-terminal part of human Scribble plays a role in the cell cycle through a mechanisms involving interaction of Scribble with a protein, Numa1, previously shown to be a key regulator of the positioning of the mitotic spindle (Nakajima et al., 2013). This function of Scribble and the interaction with Numa1 is controlled, at least in part, by phosphorylation on S1448, and could be an element of a checkpoint mechanism existing only in mammals.

It is plausible to assume that some human breast tumors are apparently able to bypass this mechanism by implementing a distinct SCRIB exon usage pattern, which results in overexpression of conserved exons encoding the N-terminal LRR domain and loss of the exons encoding the C-terminal part of the protein. Thus, loss of the apparent Scribble checkpoint function and preservation of the functions carried out by the conserved N-terminal exons appears to underpin a more malignant phenotype of breast cancer. Our results could have translational potential: if SCRIB exon usage is confirmed to have a causative effect on survival, which can be tested in cell and animal models, molecules that can affect the exon usage of SCRIB would be good candidates for developing therapies for breast cancer and assays based on measuring SCRIB exon usage can be developed as companion diagnostics for such therapies.

The intricate pattern of SCRIB exon utilization exhibited by breast tumors also raises interesting methodological questions: if other genes are subject to similar deregulation in cancer, which is a very likely proposition and possible to test, third-generation sequencing at single-molecule level, which could provide information of the frequencies of expression of transcripts containing specific exon combination, would be a necessity. This could then provide data that is likely to facilitate the development of better cancer diagnostics and therapies.

## Funding Sources

This research was supported by National Institutes of Health grant 1RO3CA150131 to MVM. We also acknowledge funding from NERC grant NE/G003688/1, on which MM was a co-investiigator.

## Conflict of Interest

The authors declare no conflict of interest.

## Authors Contributions

GM performed LC-MS/MS, transfection and imaging experiments, analyzed microscopy data and designed and performed cell cycle experiments. SA generated mutant constructs and performed wound-healing and flow cytometry experiments. BL contributed to and supervised statistical analysis. MVM designed the study, performed IP-LC/MS experiments, analyzed data, performed statistical analysis in R, and wrote the paper. All authors discussed the results and commented on the manuscript.

## Figures and Tables

**Table 1:** Patients' data for the RNA-Seq data used to calculate SCRIB exon usage statistics. The data was obtained from TCGA as described in Materials and Methods.

Phenotype/stats	No of cases	Median age in years (min-max)	Number of Node-positive tumors	Number of node-negative tumors	Number of tumors with lymph node status unavailable	Vital status = Alive	Vital status = Dead	Median survival in days (min-max)
All cases	877	59 (27–90)	393	375	109	737	140	1305 (116–7455)
ER-negative	189	53 (27–90)	70	105	14	149	40	1011 (116–7455)
ER-positive	674	60 (27–90)	316	266	92	584	90	1661 (158–6593)
ER not available	14	54 (30–87)	7	3	4	4	10	1012 (369–2534)
Her2-positive	116	62(34–90)	52	36	28	94	22	932 (116–3409)
Her2-equivocal	156	58 (27–90)	67	83	6	142	14	1050 (158–6593)
Her2-negative	465	57 (29–90)	195	211	59	415	50	1150 (197–3738)
Her2 not available or indeterminate	140	61 (27–90)	79	45	16	86	54	1869 (348–7445)
ER-Her2-	102	58 (29–90)	35	60	7	83	19	1034 (239–34,720)

**Table 2:** S1448 is required for interaction with Numa1.

Protein	WT	S1448A	S1448D	χ^2^	p-value
SCRIB	588	920	855	N/A	N/A
VIM	151	277	264	0.043	0.84
NUMA1	34	8	54	34.78	3.69E-09
GIT1	20	72	66	0	1
ARHGEF7	11	49	60	1.68	0.195

The experiment was conducted as for Supplemental Table 3 except S1448A and S1448D mutant GFP-Scribble proteins were used as baits in addition to WT Scribble. The spectral counts are from replicate LC-MS/MS runs for WT and triplicate LC-MS/MS runs for S1448A and S1448D. p-Values are calculated using the Chi squared test of independence with Yates continuity correction and the spectral counts in S1448A and S1448D columns.

## Appendix A Supplementary data

Supplementary material 1. Image 1

Supplementary material 2. Image 2

Supplementary material 3. Image 3

Supplementary material 4. Image 4
