# Supplementary material for: Decreased Usage of Specific Scrib Exons Defines a More Malignant Phenotype of Breast Cancer With Worsened Survival
Source: eBioMedicine. 2016 May 7;8:150–8. doi: 10.1016/j.ebiom.2016.05.009 (PMC4919504; doi:10.1016/j.ebiom.2016.05.009)
Supplement: Supplementary file 1 — Supplementary material 1. [file mmc1.pdf]

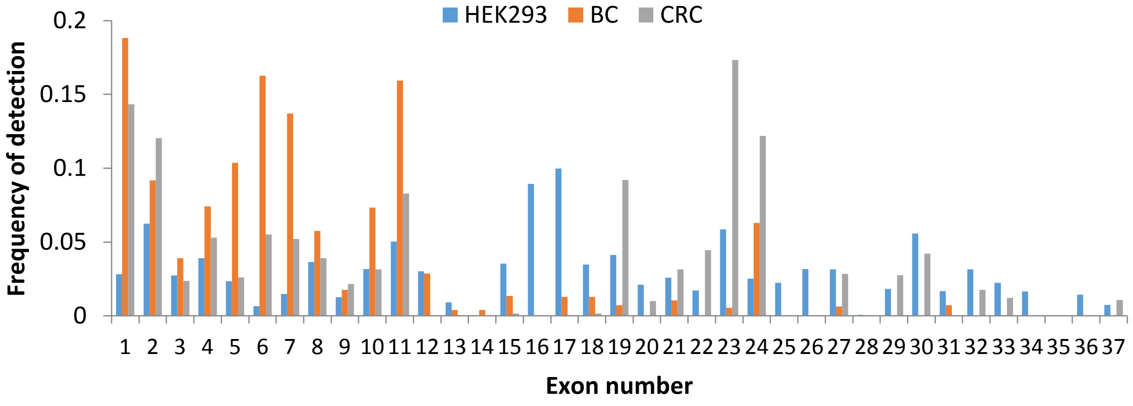

Supplemental Fig. 1. Frequency of detection of Scribble peptides encoded by specific exons in IP-LC/MS experiments with HEK293 cells (n=9), colorectal tumors (n=19), and membrane protein fractions from breast cancer samples (n=62). HEK293 cells were transfected with a plasmid encoding GFP fusion of wild type Scribble and various mutants. A monoclonal anti-GFP antibody was then used to immunoprecipitate the GFP-Scribble proteins. The precipitated proteins were digested with trypsin and analyzed by high-resolution mass spectrometry. Frozen breast tumors (n=62) were used to isolate membrane-associated proteins, which were digested and analyzed as the samples obtained from transfected HEK293 cells.

The contingency table below gives the summed up spectral counts for the N-terminal exons 4 to 6 and for the C-terminal exons 31 to 33 for HEK293 and breast tumor samples.

	HEK	BC
N-term (4-6)	194	299
C-term (31-33)	258	9

χ^2 test of independence gives a p-value of 1.09e-52.

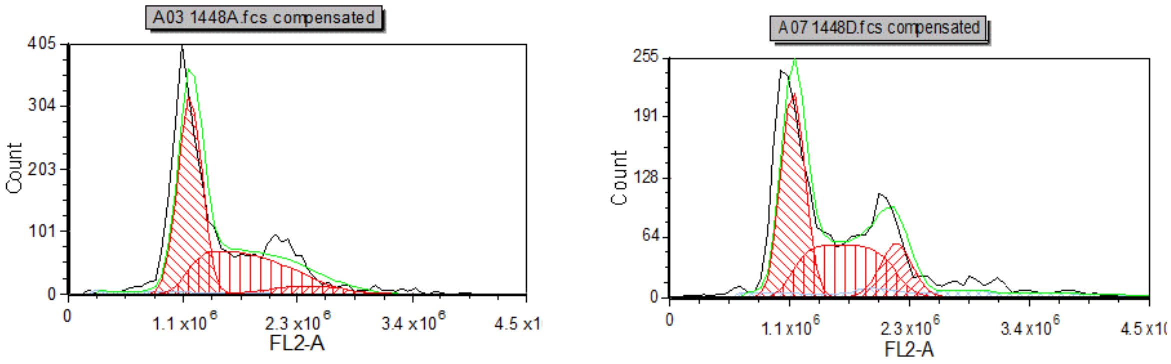

Cycle	G1 Mean	G1 CV	%G1	G2 Mean	G2 CV	%G2	%S	G2/G1	%Total	B.A.D.
Diploid	1199656.14	9.96	51.07	2363319.91	15.86	6.62	42.31	1.97	100.00	1.50

Cycle	G1 Mean	G1 CV	%G1	G2 Mean	G2 CV	%G2	%S	G2/G1	%Total	B.A.D.
Diploid	1164902.40	9.52	44.73	2138787.02	7.01	16.06	39.21	1.84	100.00	6.01

Supplemental Fig. 2. Cell cycle analysis. Gated histograms with a model of the distribution of FITC++PI+ HEK293T cells transfected with S1448A (left) and S1448D (right) mutants of Scribble. The same gating was applied to each sample and adjusted to find the model to fit their histograms. The x-axis (FL2-A) represents the total fluorescence intensity measured by the channel detecting PI-stained DNA of cell population that were positively expressing the respective GFP-Scribble. The y-axis (count) represents the number of cells under each cell cycle phase depicted by three red-patterned curves under the histogram. These results are representative for 4 independent transfection experiments.

Supplemental Table 1: Scribble peptides detected by high-resolution LC-MS/MS in experiments with breast tumors (n=62), colorectal tumors (n=19), and transfected HEK293 cells (n=9). The numbers in the last 3 columns represent spectral counts.

Sequence	Position	Exon	HEK293	CRC	BC
HVESVDKR	13-20	1	18	0	0
HCSLQAVPEEIYR	21-33	1	40	0	0
SLEELLLDANQLR	37-49	1	54	187	236
ELPKPFFR	50-57	1+2	8	0	0
KLGLSDNEIQR	63-73	2	33	0	0
LGLSDNEIQR	64-73	2	24	67	87
LPPEVANFMQLVELDVSR	74-91	2	135	90	28
LPPEVANFMQLVELDVSRNDIPEIPESIK	74-102	2+3	40	0	0
NDIPEIPESIK	92-102	2+3	26	0	0
ALEIADFSGNPLSR	106-119	3+4	50	31	49
LPDGFTQLR	120-128	4	44	31	1
SLAHLALNDVSLQALPGDVGNLANLVTLELR	129-159	4+5	72	7	43
SLPASLSFLVK	165-175	5+6	28	27	87
LEQLDLGGNDLEVLPDTLGALPNLR	176-200	6+7	0	45	117
NQLSALPPELGNLR	207-220	7+8	43	23	55
NQLSALPPELGNLRR	207-221	7+8	20	0	0
LVCLDVSENR	222-231	8	21	0	17
LEELPAELGGLVLLTDLLLSQNLLR	232-256	8	17	0	0
RLPDGIGQLK	257-266	8+9	32	28	0
LPDGIGQLK	258-266	8+9	22	0	0
LCEVTEAIGDCENLSELILTENLLMALPR	278-306	9+10	0	0	22
LTNLNVDR	314-321	10	23	23	27
LTNLNVDRNHLEALPPEIGGCVALSVLSLR	314-343	10	2	0	0
NHLEALPPEIGGCVALSVLSLR	322-343	10	48	0	43
LAVLPPELAHTTELHVLDVAGNR	347-369	10+11	62	18	0
LQSLPFALTHLNLK	370-383	11	53	69	111
LQSLPFALTHLNLKALWLAENQAQPMLR	370-397	11	22	0	0
ALWLAENQAQPMLR	384-397	11	59	20	71
FQTEDDAR	398-405	11	18	1	18
VSVIQFLEAPIGDEDAEEAAAEK	445-467	12	60	0	36
VSVIQFLEAPIGDEDAEEAAAEKR	445-468	12	68	0	0
RATPHPSELK	473-482	13	19	0	0
ATPHPSELK	474-482	13	20	0	0
RSEACPCQPDSGSPLPAEEEKR	492-513	13+14	0	0	5
EIEEGQPEAPWTLPGGR	583-599	15	40	2	17
LPQPEAVVALLQGMQPDGEGPVAPGGWHNGPHAPWAPR	619-656	15	96	0	0
EGAVVSAPSVK	695-705	15	14	0	0
GVSFDQANNLLIEPAR	706-721	16	50	0	0
GVSFDQANNLLIEPARIEEEELTLTILR	706-733	16+17	201	0	0
IEEEELTLTILR	722-733	16+17	129	0	0
QTGGLGISIAGGK	734-746	17	24	0	0
GSTPYKGDDEGIFISR	747-762	17+18	49	0	0
GDDEGIFISR	753-762	17+18	21	0	16

VSEEGPAAR	763-771	18	16	1	0
LLEVNGVALQGAEHHEAVEALR	780-801	18+19	62	1	0
GAGTAVQMR	802-810	19	23	0	0
MVEPENAVTITPLRPEDDYSPR	816-837	19	0	8	9
LPLLPPESPGPLR	846-858	19	45	74	0
GLGFSIAGGK	871-880	19	19	37	0
AGDAGIFVSR	887-896	19+20	26	0	0
IAEGGAAHR	897-905	20	8	0	0
AGTLQVGDR	906-914	20	21	0	0
VLSINGVDVTEAR	915-927	20+21	35	13	0
HDHAVSLLTAASPTIALLLER	928-948	21	75	28	13
AGGPLGLSIVGGSDHSSHPFGVQEPGVFISK	1010-1040	22	73	58	0
ILAVNGQDVR	1058-1067	23	24	159	3
DATHQEAVSALLR	1068-1080	23	37	0	0
DATHQEAVSALLRPCLELSLLVR	1068-1090	23	47	0	0
PCLELSLLVR	1081-1090	23	12	0	0
RDPAPPGLR	1091-1099	23	16	0	0
DPAPPGLR	1092-1099	23	15	0	0
GHAGNPRDPTDEGIFISK	1121-1138	23	62	0	0
DPTDEGIFISK	1128-1138	23	36	67	4
VSPTGAAGR	1139-1147	24	2	0	0
LLEVNQQSLLGLTHGEAVQLLR	1157-1178	24	76	159	79
SVGDTLTVLVCDGFEASTDAALEVSPGVIANPFAAGIGHR	1179-1218	24+25	29	0	0
NSLESISSIDR	1219-1229	25	24	0	0
NSLESISSIDRELSPEGPGK	1219-1238	25	42	0	0
NSLESISSIDRELSPEGPGKEK	1219-1240	26	17	0	0
ELSPEGPGK	1230-1238	26	22	0	0
ELSPEGPGKEK	1230-1240	26	8	0	0
EKELPGQTLHWGPEATEAAGR	1239-1259	26+27	42	0	0
ELPGQTLHWGPEATEAAGR	1241-1259	26+27	46	0	0
ALAAVPSAGSVQR	1270-1282	27	46	37	8
MAESPCSPSGQQPPSPPSPDELPANVK	1292-1318	28+29	3	0	0
AFAAVPTSHPPEDAPAQPPTPGPAASPEQLSFR	1323-1355	29+30	75	36	0
QKYFELEVR	1358-1366	30	31	0	0
YFELEVR	1360-1366	30	28	3	0
VPQAEGPPK	1367-1375	30	21	0	0
VPQAEGPPKR	1367-1376	30	20	0	0
RVSLVGADDLR	1376-1386	30	14	16	0
RVSLVGADDLRK	1376-1387	30	3	0	0
VSLVGADDLR	1377-1386	30	24	0	0
VSLVGADDLRK	1377-1387	30	21	0	0
EAAEAGAEAR	1406-1415	31	14	0	9
LALDGETLGEEEQEDEQPPWASPSPTSR	1416-1443	31+32	57	0	0
QSPASPPPLGGGAPVR	1444-1459	32	29	0	0
LRVQSPEPPAPER	1471-1483	32	2	0	0
VQSPEPPAPER	1473-1483	32	29	23	0
ALSPAELR	1484-1491	32	17	0	0
SLEQDALR	1508-1515	33	27	16	0

AQMVLSR	1516-1522	33	20	0	0
LAEAPSPAPTPSPTPVEDLGPQTSTSPGR	1536-1564	33+34	48	0	0
LSPDFAEELR	1565-1574	34	22	0	0
SLEPSPSPGPQEEDGEVALVLLGR	1575-1598	36	61	0	0
PSPGAVGPEDVALCSSR	1599-1615	37	30	14	0
SLEPSPSPGPQEEDGEVALVLLGRPSPGAVGPEDVALCSSR	1575-1615	37	2	0	0

Supplemental Table 2. Identification of Scribble-interacting proteins by IP-LC/MS.

Protein	GFP	Scribble	G-test	p-value	Adj. p
SCRIB	0	704	1296.242	7.4E-284	7.3E-281
VIM	82	221	134.2677	4.77E-31	4.68E-28
NUMA1	1	49	76.34397	2.38E-18	2.34E-15
GIT1	0	37	61.05041	5.56E-15	5.45E-12
NEFM	3	40	51.91171	5.81E-13	5.69E-10
LTF	4	38	45.12518	1.85E-11	1.81E-08
INA	0	33	53.96582	2.04E-13	2E-10
ARHGEF7	0	26	41.64128	1.1E-10	1.07E-07
ENO1	9	27	17.53443	2.82E-05	0.027651
GIT2	0	17	26.01244	3.39E-07	0.000332
NOS1AP	0	14	20.89427	4.85E-06	0.004756
PIGR	1	15	18.66901	1.55E-05	0.015238
TRIM28	2	16	17.16998	3.42E-05	0.033496
ZNF281	0	13	19.20386	1.17E-05	0.011513
SUGT1	0	12	17.52309	2.84E-05	0.027816

Protein	WT	Δ C-term	χ^2	p-value
SCRIB	588	405	N/A	N/A
VIM	151	17	57.09	4.17E-14
NUMA1	34	0	21.22	4.09E-06
GIT1	20	54	27.81	1.34E-07
ARHGEF7	11	61	50.89	9.77E-13

Top: Identification of Scribble-interacting proteins by IP-LC/MS. Plasmids encoding GFP-Scribble or GFP as a negative control were used to transfect HEK293 cells and the expressed proteins were immunoprecipitated using a mouse monoclonal anti-GFP antibody, digested and analyzed by LC-MS/MS. The spectral counts in column 2 and 3 are from 3 replicate LC-MS/MS runs. The p-values are calculated by G-test and adjusted by the Bonferroni method. **Bottom**: The C-terminus of Scribble is required for interaction with Vimentin and Numa1. The experiment was conducted as for the top part except a truncated GFP-Scribble protein was used as bait and compared to wild type Scribble. The spectral counts are from duplicate LC-MS/MS runs for WT and triplicate for Δ C-term. P-values are calculated using the Chi squared test of independence with Yates continuity correction.

Supplemental Table 3. Wound healing assays with WT Scribble and S1448A and S1448D mutants. The assays were performed as described in Materials and Methods in 4 replicated transfections.

Wound area data

	**0 hr**				**24 hr**			
**Transfection**	**1**	**2**	**3**	**4**	**1**	**2**	**3**	**4**
**S1448A**	9.794	10.921	9.366	9.752	9.492	10.687	9.023	9.370
**S1448D**	13.081	11.314	10.167	16.932	12.943	11.116	9.922	16.802
**WT**	12.543	12.060	12.199	13.356	12.318	11.931	12.046	13.220

Pair wise p-values

	**S1448A**	**S1448D**	**WT**
**S1448A**	N/A	0.0194	0.0044
**S1448D**		N/A	
**WT**		0.623	N/A

Supplementary R script 1. The file scrib_revised.html contains an R script which can be used together with Supplementary Dataset 1 to reproduce the analysis of SCRIB exon expression and survival in breast cancer.

Supplementary Dataset 1. The file scrib_exons_clinical_full.csv contains the RPKM counts for SCRIB exons and clinical information, which can be used together with R script 1 to reproduce the analysis of SCRIB exon expression and survival in breast cancer.

Supplementary Dataset 2. The file MCF10A_scrib_exon_norm.csv contains the RPKM counts for SCRIB exons in MCF10A cells, which can be used together with R script 1 to reproduce the analysis of SCRIB exon expression in MCF10A cells.
