# Supplementary material for: Decreased Usage of Specific Scrib Exons Defines a More Malignant Phenotype of Breast Cancer With Worsened Survival
Source: eBioMedicine. 2016 May 7;8:150–8. doi: 10.1016/j.ebiom.2016.05.009 (PMC4919504; doi:10.1016/j.ebiom.2016.05.009)
Supplement: Supplementary file 4 — Supplementary material 4. [file mmc4.zip › EBI00602-mmc4.html]

scrib\_revised


 


In [1]:

```
########################################################################################
#######  Survival analysis of scribble exon expression in breast cancer
#######  Metodi V. Metodiev, University of Essex, July 2015
#######  RNA-seq data from TCGA are used. The individual files are merged with
#######  clinical data and assembled into a dataframe and exported to csv file called
#######  "scrib_exons_clinical_full.csv"
########################################################################################

#Read data from file
data.subset<-read.csv("scrib_exons_clinical_full.csv", stringsAsFactors=F)
#Create a vector with summed scribble exon RPKM counts to be used for normalization
scrib<-apply(data.subset[,-c(1:129)], 1, sum)
```

In [2]:

```
#Load survival package, which needs to be pre-installed, and create survival and
#vital vectors
library(survival)
survival<-as.numeric(ifelse(data.subset$days_to_last_followup!="[Not Available]",
data.subset$days_to_last_followup, data.subset$days_to_death))
vital<-ifelse(data.subset$vital_status!="Dead", 0,1)
surv1<- Surv(survival, vital)
```

In [3]:

```
#Normalize exon counts against sum of scribble in other exons
exon.norm<-apply(data.subset[,-c(1:129)], 2, function(x) x/(scrib-x))
exon.norm<-exon.norm[,36:1]
colnames(exon.norm)<- c(1:29, "30/31", 32:37)
```

In [13]:

```
#Survival analysis on clustered data
clust1<-kmeans(exon.norm, 4)
survdiff(surv1~clust1$cluster)
plot(survfit(surv1~clust1$cluster), col=clust1$cluster,
	xlim=c(0,5000))
```

Out[13]:

```
Call:
survdiff(formula = surv1 ~ clust1$cluster)

                   N Observed Expected (O-E)^2/E (O-E)^2/V
clust1$cluster=1 424       58    66.57     1.103     2.152
clust1$cluster=2 172       28    29.92     0.123     0.157
clust1$cluster=3 239       52    35.86     7.267    10.140
clust1$cluster=4  42        2     7.66     4.182     4.444

 Chisq= 13  on 3 degrees of freedom, p= 0.00456
```

In [15]:

```
#Two clusters have nearly identical survival curves so we merge them
#Keep in mind that cluster numbering may differ between runs
class<-ifelse(clust1$cluster==2,1,clust1$cluster)
survdiff(surv1~class)
```

Out[15]:

```
Call:
survdiff(formula = surv1 ~ class)

          N Observed Expected (O-E)^2/E (O-E)^2/V
class=1 596       86    96.48      1.14      3.78
class=3 239       52    35.86      7.27     10.14
class=4  42        2     7.66      4.18      4.44

 Chisq= 12.9  on 2 degrees of freedom, p= 0.00155
```

In [16]:

```
plot(survfit(surv1~class), col=c("grey", "red", "blue"),
	xlim=c(0,5000), xlab="Time to death (days)", ylab="Survival")
text(950, 0.05, "p= 0.00155", cex=0.85)
```

In [27]:

```
rpkm<-read.csv("MCF10A_scrib_exon_norm.csv", stringsAsFactors=F)
x<-c(1:35)
x0<-x+0.4
x2<-c(2:36)
low.risk<-boxplot(exon.norm[class==4,])
high.risk<-boxplot(exon.norm[class==3,])
med.risk<-boxplot(exon.norm[class==1,])
mcf10a<- boxplot(rpkm.norm)
```

In [28]:

```
boxplot(100*exon.norm[class==4,], outline=F, border="blue", boxlty = 3,
	whisklty = 0, staplelty = 0, ylim=c(0,20), cex.axis=.75, xlab="SCRIB exons",
	ylab="Exon usage (%)", notch=F)
segments(x0,100*low.risk$stats[c(3),1:35],x1=x2-0.4,100*low.risk$stats[c(3),2:36],
	col="blue")

boxplot(100*exon.norm[class==3,], add=T, outline=F, border="red", boxlty = 3,
	whisklty = 0, staplelty = 0, cex.axis=.75, notch=F)
segments(x0,100*high.risk$stats[c(3),1:35],x1=x2-0.4,100*high.risk$stats[c(3),2:36],
	col="red")

boxplot(100*exon.norm[class==1,], add=T, outline=F, border="dark grey", boxlty = 3,
	whisklty = 0, staplelty = 0, cex.axis=.75, notch=F)
segments(x0,100*med.risk$stats[c(3),1:35],x1=x2-0.4,100*med.risk$stats[c(3),2:36],
	col="dark grey")

boxplot(100*rpkm.norm, add=T, outline=F, border="dark green", boxlty = 3,
	whisklty = 0, staplelty = 0, cex.axis=.75, notch=F)
segments(x0,100*mcf10a$stats[c(3),1:35],x1=x2-0.4,100*mcf10a$stats[c(3),2:36],
	col="dark green")

legend(1, 20, legend=c("Low risk", "Medium risk", "High risk", "MCF10A"),
       col=c("blue", "dark grey", "red", "dark green"), lty=1, cex=0.8)
```

In [29]:

```
library(gplots)
```

In [30]:

```
heatmap.2(scale(exon.norm), col=redblue(256), scale="row", key=T, keysize=1.5,
          density.info="none", trace="none",cexCol=0.9, labRow=NA, 
		  hclustfun= function(x) hclust(x, method="ward.D"))
```

In [31]:

```
library(fpc)
plotcluster(exon.norm, class)
```

In [ ]:
